# Enhancing Nucleation using a Vortex-based Hydrodynamic Cavitation Device: Application to Antisolvent Crystallization of Paracetamol – Methanol – Water System

**DOI:** 10.1016/j.ultsonch.2025.107668

**Published:** 2025-11-11

**Authors:** Vidit Tiwari, Subhrajit Swain, Vivek V. Ranade

**Affiliations:** Multiphase Reactors and Intensification Group Synthesis and Solid-State Pharmaceutical Research Centre Bernal Institute, University of Limerick, Ireland

**Keywords:** Continuous nucleator, Induction time, COBC, CSD, Yield, Productivity

## Abstract

Nucleation is the onset of the crystallization process, and enhancing its rate is fundamental to intensifying crystallization, be it in a batch or continuous mode of operation. The present work investigates, for the first time, the influence of hydrodynamic cavitation (HC) on enhancing the nucleation rate of paracetamol crystallization in a methanol–water system. Initially, the experiments were performed in a batch mode to determine the induction time using a vortex-based HC device (VD). The experimentally determined induction time was found to decrease with supersaturation. The use of HC was found to enhance nucleation and significantly reduce induction time. An appropriate correlation to estimate induction time is developed. Based on these findings, the VD-based continuous nucleator using a loop configuration was developed to intensify the performance of a continuous oscillatory baffle crystallizer (COBC). The VD-based nucleator improved the yield and productivity of the COBC, significantly reducing the risk of encrustation and clogging in the bends of the COBC. The approach and results presented in the current study will provide a sound basis for harnessing VD-based hydrodynamic cavitation for enhancing nucleation and intensifying antisolvent crystallization.

## Highlights

• Demonstrated use of Hydrodynamic Cavitation (HC) to enhance nucleation for the first time.
• Developed a correlation to quantify the effect of HC on induction time.
• HC assisted nucleation led to significant reduction in encrustation and fouling.
• Use of HC assisted pre-nucleator before continuous tubular crystallizer improved yield and productivity.

## 1 Introduction

Over 90 % of the manufacturing processes of small molecule Active Pharmaceutical Ingredients (APIs) involve crystallization, which is an essential unit separation and purification technique used in API manufacturing [1]. Control over the crystal’s critical quality attributes (CQAs), such as crystal size, shape, polymorph, etc, is necessary to improve the product’s bioavailability, processability, and overall performance. Continuous crystallization has emerged as a promising technology that provides benefits such as high productivity, better control over crystallization mechanisms (nucleation and growth), and reduced capital and operational costs. The development of a continuous crystallization process involves careful selection of the solvent system, a suitable approach to generate supersaturation (cooling, antisolvent addition, evaporation), and controlling process parameters such as antisolvent addition rate, cooling rate, and mixing [2]. However, solvent selection is often constrained by upstream requirements [3,4], making the control of nucleation and growth even more challenging.

When CQA requirements like size and shape cannot be achieved, alternative approaches like continuous seeding [[5], [6], [7]], additives [[8], [9], [10]], and the use of in-line devices [[11], [12], [13], [14], [15], [16], [17]] are explored. Continuous seeding requires a consistent quality of seeds, which is difficult to achieve without in-situ generation of seeds. Additives may get incorporated into the crystal lattice and may adversely affect the product purity [18]. Considering this, an ability to modify nucleation rate will be desirable for tailoring CQAs. Nucleation, where molecular clusters form and self-assemble into a structured array, leading to the development of a crystal lattice [19], is the first step of crystallization. Nucleation is governed by supersaturation, determined by concentration and solubility [20]. However, other factors such as impurities, agitation, shear, and cavitation [[21], [22], [23]] also affect the nucleation rate. Shear affects nucleation by enhancing mass transport at intermediate shear rates or causing breakup of molecular clusters, which are precursors to nuclei, at high shear rates [24]. Additionally, factors like crystallizer volume and confinement can alter local shear conditions and surface interactions, impacting nucleation kinetics and crystal formation [25]. Because of this, high-shear wet mills are used to enhance nucleation in continuous crystallization processes [11,26]. However, the lack of any specific scale-up/down criteria, high energy demands, and a lower limit to particle size make the use of wet mills challenging across different scales [27]. Ultrasonic or acoustic cavitation (AC) has been used to tailor crystallization processes by expediting nucleation [28] and, thereby, gaining control over CQAs like crystal size distribution [29] (CSD) and polymorphic forms [23]. However, AC has its drawbacks, such as probe surface erosion leading to product contamination [13] and limited amenability to scale-up. A recent study on the nanobubble-assisted crystallization of glycine [30] shows that bubbles act as nucleation sites, leading to a faster nucleation rate in antisolvent crystallization. In this work, we present hydrodynamic cavitation (HC) as a potential tool to enhance nucleation rates for antisolvent crystallization for the first time.

HC is the generation, growth, and collapse of vapour bubbles or cavities due to pressure changes in a flowing liquid. HC has benefits over AC and wet mills, such as no moving parts, low energy consumption [31] and scalability [32]. In this work, we have used the vortex-based HC device (VD), which exhibits early inception of cavitation and long working life by shielding the device surfaces from cavitation [33]. Previous studies have shown the applicability of HC to crystallization [32,34] and particle breakage [31,35]. However, applying HC to enhance the nucleation rate of a crystallization process is demonstrated in this work for the first time. In this work, the effect of HC on nucleation kinetics during the antisolvent crystallization of paracetamol (PCM) is quantified through a set of batch experiments conducted in methanol–water solution [36]. Typically, measurements of Metastable Zone Width (MSZW)[37] or induction time ($t_{i}$)[38] are used for quantifying the primary nucleation process. Measurement of MSZW for antisolvent crystallization (defined as the excess antisolvent composition at which the number density of detectable primary nuclei reaches a definite value [39]) is challenging since it depends on a number of factors, such as placement of the antisolvent addition pipe, addition rate, time taken for addition to complete, operational rate of the pump employed, and placement of the probe (along with the detection limit of the probe)[36]. Therefore, in this work, we have used measurements of the induction time (defined as the time taken for the number of detectable crystals to reach a certain pre-defined critical value, $t_{i}$) for quantifying the influence of HC on the nucleation. After establishing the efficacy of HC for enhancing nucleation, we have demonstrated the use of the vortex-based HC device (Vortex Diode, VD) for enhancing nucleation for the continuous antisolvent crystallization conducted in a tubular crystallizer (Continuous Oscillatory Baffled Crystallizer, COBC). The VD-based nucleator is shown to improve the productivity and yield of COBC. It also reduced the risk of clogging and encrustation in the bends used in the COBC. The approach and results presented in this work will provide a sound basis for further applications of HC-assisted crystallization or cavi-crystallization.

## 2 Methodology

### 2.1 Materials

Paracetamol (98 % pure) was obtained from Thermo Scientific Chemicals, and Methanol (≥99.9 %, HPLC grade) was sourced from Merck Life Science Limited. Deionized (DI) water was drawn using the Elga PureLab (conductivity ∼ 1µS/cm). Whatman 6 (retention size: 3 µm) filter papers were obtained from SLS Scientific Laboratory Supplies (Ireland) Limited.

### 2.2 Setup

**Fig. 1:**
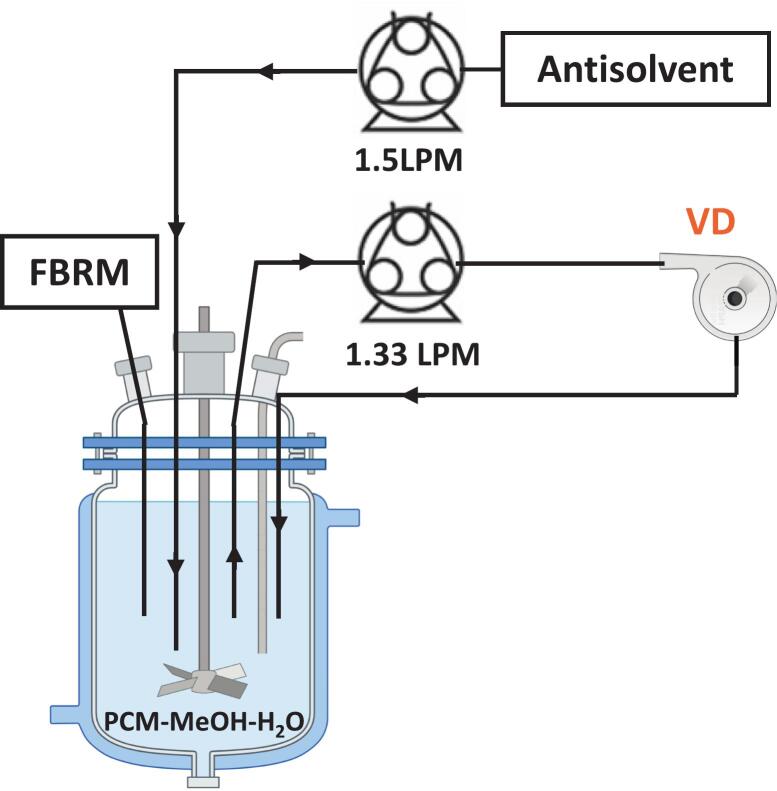
An experimental setup for the induction time measurement experiments.

**Fig. 2:**
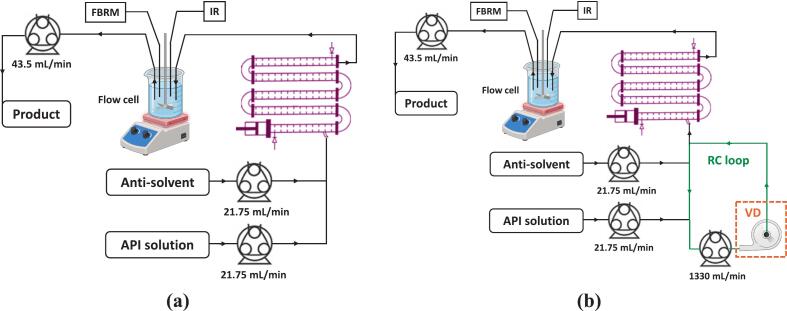
Experimental setup for continuous antisolvent crystallization of paracetamol in a **(a)** COBC, **(b)** RC + COBC (without the VD shown in box of orange dashed lines), and VD + RC + COBC configurations.

The experimental setups used in this work are shown schematically in Fig. 1, Fig. 2. For the induction time measurement experiments, the setup (Fig. 1) comprised an Optimax^TM^ (Mettler Toledo) reactor system equipped with an overhead stirrer connected to a four-blade 45° downward-pitched impeller, and a Teflon reactor head. The stirrer speed was set at 350 rpm, which is higher than the critical suspension speed (∼270 rpm) but low enough to avoid any crystal breakage [34,40]. The critical suspension speed was estimated using the Zwietering correlation and relevant physical properties of the system (refer to **Section S1** of the SI). The antisolvent stream was introduced near the high-velocity zone between the stirrer shaft and the impeller tip using an appropriately programmed Longer L100-1F Intelligent Pump (Precision Fluid Transfer) fitted with a YZ1515x pump head and Longer #18 silicone tubing (ID: 7.9 mm, OD: 11.1 mm) at a flow rate of 1.5 L/min (LPM) to avoid local supersaturation. More details on the estimation of the volumetric flow rate and programming of the intelligent pump can be found in **Section S1** of the SI. A vortex-based HC device (VD) with a nominal flow rate of 1.33 LPM [see Simpson and Ranade (2019)[41] for more details of device design and dimensions] was utilized in the loop using a peristaltic pump (Longer WT600-2 J) with a KZ25 pump head and Longer #35 (ID: 7.9 mm, OD: 12.7 mm) silicone tubing. The extent of cavitation realized in the cavitation device is a function of the pressure drop across the device [42]. Previous studies indicated that cavitation inception occurs at a pressure drop of less than 100 kPa [43]. Previous studies on droplet breakage [44], particle breakage [31,45] and pollutant degradation [46,47] indicated that the effective and safe operating range of pressure drop across VD is between 100 kPa and 250 kPa. In this work, we, therefore, operated the HC device with a pressure drop of 175 kPa (corresponding to a flow rate of 1.33 LPM). The number count of the particles was tracked using an in-line Focused Beam Reflectance Measurement (FBRM, Mettler Toledo^TM^) G400 probe. The lifetime of cavities generated by HC is less than 1 ms [33]. The transit time from the outlet of the cavitation device to the inlet of the stirred tank is 2800 ms. Therefore, the cavities generated in a vortex device are unlikely to reach the stirred tank and did not interfere with the counts detected by FBRM. The probe measured the total number of particles, with data recorded every 10 s at a scan speed of 2 m/s [48]. A vacuum filtration assembly comprising a KNF LABOPORT® N 820.3 FT.18 diaphragm pump, Büchner funnel (90 mm), and Whatman 6 filter paper was used to collect solids for characterization.

For evaluating the potential of VD-based enhancement of nucleation for intensifying continuous antisolvent crystallization, a VD-based continuous nucleator was designed. In continuous stirred tank crystallizers (CSTCs), a significant number of crystals are always present in the crystallizer at the steady state, and therefore, the influence of the nucleator coupled to fully mixed CSTCs is not expected to be significant. Whereas, in a tubular crystallizer used for conducting unseeded antisolvent crystallization, because of less back mixing than CSTC, a significant portion of the tubular crystallizer is used for nucleation rather than for growth. For such cases, using a nucleator with enhanced nucleation via VD can significantly improve overall performance and productivity. In order to evaluate this potential, an experimental setup for continuous crystallization of paracetamol is established using a continuous oscillatory baffled crystallizer (COBC). The setup is shown schematically in Fig. 2. The setup consisted of a COBC (NiTech DN15 LITE) comprising five straight tubes (of length 0.7 m, comprising 26 baffles each) and four connecting bends (6 baffles each) with a total volume of 547 mL. A loop configuration, as shown in Fig. 2b was used as a continuous nucleator (a labelled photograph of the arrangement is shown in Fig. S2).

Experiments were conducted with the following three configurations for facilitating a systematic comparison of the influence of the VD-assisted nucleator on overall performance:

a) **COBC:** Conventional COBC with the addition of feed streams at the inlet of COBC, followed by a flow cell.
b) **RC + COBC:** COBC with the feed streams injected into a loop (functioning as a CSTC) before passing on to the inlet of COBC (n-tanks-in-series), followed by a flow cell (∼CSTC).
c) **VD + RC + COBC:** COBC with the feed streams injected into a loop comprising VD (functioning as a CSTC) before passing on to the inlet of COBC (n-tanks-in-series), followed by a flow cell (∼CSTC).

The three cases were selected to represent (a) a conventional COBC, (b) a COBC with a continuous nucleator without VD-based cavitation before the inlet, and (c) an augmented COBC with a loop similar to (b) but with a VD for enhancing nucleation via HC. Other components of the setup include a Julabo® open circulating bath, which was used to maintain the crystallizer temperature at 25 °C. Longer L100-1F Intelligent (Precision Fluid Transfer) pumps were employed with YZ1515x pump heads and Longer #18 (ID: 7.9 mm, OD: 11.1 mm) silicone tubing to inject the feed streams (the API solution and the antisolvent). A Longer WT600-2 J pump with a KZ25 head and Longer #35 (ID: 7.9 mm, OD: 12.7 mm) tubing was used for the recirculation loop. Paracetamol was dissolved in methanol and DI water to prepare the API solution, such that the solute-free concentration of paracetamol ($C_{S}$) and methanol in the API solution stream were 0.1 g Solute/g Solvents and 0.37 g methanol/g Solvents, respectively. Pure DI water was utilized as the antisolvent. Both the feed streams were maintained at 25 °C using feedback temperature control, utilizing heating plates (IKA^TM^) and temperature probes (IKA^TM^ PT1000.60). A flow cell (∼80 mL volume, housed within an isothermal water bath and maintained at 25.0 ± 0.1 °C using SLS Lab Basics 280C Hotplate Stirrer with PT1000 probe) at the outlet of the COBC was used to place the FBRM probe for tracking chord length distribution (CLD), and an ATR-FTIR probe (React IR, Mettler Toledo^TM^) equipped with a 9.5 mm “DiComp” immersion probe with a diamond crystal and silver halide (AgX) optical cable to track the concentration of dissolved paracetamol in the crystallization mixture. An overhead stirrer (Heidolph Hei-TORQUE 200 Precision Base, Germany) equipped with a 45° downward-pitched-blade impeller with four blades was used to stir the solution in the flow cell at 250 rpm. Product was taken out using a Longer BT300-2 J, fitted with a YZ1515x pump head and Longer #16 (ID: 3.2 mm, OD: 6.4 mm) tubing. Considering the short residence time in the flow cell (1.8 mins), small area exposed to the atmosphere (16.7 cm^2^), and maintained temperature ensured that there is minimal influence of not enclosing the top of the flow cell, and therefore, the flow cell top was not covered in the present work (Fig. S2).

### 2.3 Procedure and data Analysis

The induction time measurement experiments were started by filling the Optimax reactor with the API and the solvents (methanol and DI water) to prepare the API solution. The composition and quantities of the API solution and antisolvent used in the induction time measurement experiments are listed in. The reactor was heated to 30 °C to ensure complete dissolution of the API and then cooled to 25 °C. The temperature was maintained at 25 °C. A speck-free solution was ensured before the start of each experiment by confirming zero counts (0 ± 5) from the FBRM. All the experiments were conducted with and without incorporating the VD into the loop across four different supersaturations (S), ranging from 0.1 to 0.6, which is defined as,

(1) $$S=\frac{C}{C^{*}}-1$$

where C is the solute-free concentration (g Solute/g Solvents) of the solute in the crystallizing mixture (API solution + antisolvent), and $C^{*}$ is the solute-free solubility (g Solute/g Solvents) of the solute in terms of mass fraction in the crystallizing mixture. Regarding the influence of composition on cavitation, the extent of cavitation for a given device and operating pressure drop is governed primarily by the viscosity and vapor pressure of the mixture [49]. Over the range of compositions considered in this work, both these physical properties do not change significantly, and therefore, the extent of cavitation is not expected to vary significantly with the change in composition (methanol to water ratio). However, for the same supersaturation, S, changes in the methanol mass fraction would need a significant change in the paracetamol concentration. For example, when the final antisolvent mass fraction (x_w_) changes from 0.73 to 0.65 at S=0.4, the initial paracetamol concentration would change from 0.075 to 0.126 g/g Solvents (Table 1). This change in initial paracetamol concentration may influence the measured induction time (a higher concentration is expected to reduce induction time). Therefore, in this work, all the experiments (across different supersaturations) were carried out at a constant solvent composition (x_w_ = 0.73) to ensure a fair comparison across the range of supersaturations with/ without HC. The fixed amount of antisolvent (400 ± 2 mL) was added using the appropriately programmed intelligent pump at a volumetric flow rate of 1.5 LPM, corresponding to a feed time of ∼ 16 s. The mixing time for such a system can be estimated to be ∼ 2 s (**Section S1** of the SI). The range of S was chosen such that the induction time is at least an order of magnitude greater than the feed and mixing time. All the experiments across this range of S were carried out in triplicate to quantify reproducibility and error bars.

**Table 1:** Composition and quantities used in the induction time experiments. All the mass fractions and concentrations reported are on a solute-free basis.

**Experiment name**		**COMP**	**SS1**	**SS2**	**SS3**	**SS4**
**Supersaturation,**S**(−)**		0.4	0.6	0.4	0.2	0.1
**API Solution**	PCM (g)	107	64.4	56.3	48.3	44.3
	Methanol (g)	300	200			
	Water (g)	150	150			
**Antisolvent (AS)**	Water (g)	550	400			
**Final AS mass fraction, x_w_**		0.65	0.73			
**Initial concentration, C (g/g Solvents)**		0.126	0.086	0.075	0.064	0.059
Solubility, C* (g/g Solvents)		0.09	0.054			
**Pressure drop across VD,** Δ**P (kPa)**		175				

**Fig. 3:**
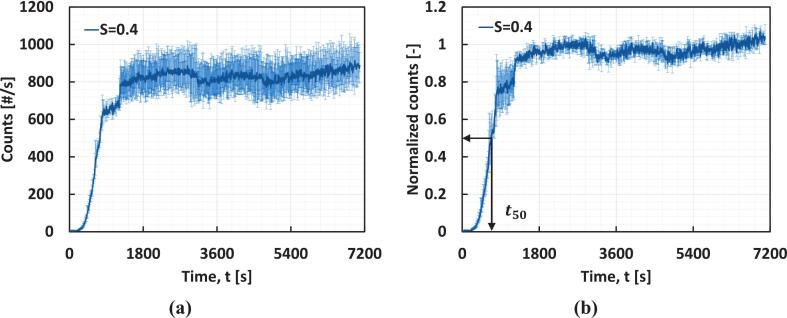
**(a)** Evolution of FBRM counts with time at a supersaturation S=0.4, **(b)** Normalization of the counts v/s time data to define the quantile times at S=0.4. The error bars represent the range between the minimum and maximum values obtained from three independent experiments, plotted around the average values.

The experiments were conducted for at least 1800 s beyond the point at which the number of counts detected by FBRM plateaued. FBRM probes typically measure chord lengths from approximately 1 to 1000 µm. During data processing, all raw FBRM counts were used as recorded without applying additional thresholds, meaning the effective lower bound for detection is governed by the probe’s hardware limit of ∼ 1 µm. For comparison between the case with and without VD, the counts per second data were normalized. Fig. 3a shows the counts v/s time during a typical batch antisolvent crystallization of paracetamol. To normalize the measured counts, the maximum counts were identified by averaging the counts after they had plateaued. The measured counts were then divided by the maximum counts. Fig. 3b shows the normalized counts v/s time data. A similar procedure was used to normalize the counts for all the batch experiments. The 10 %, 50 % and 90 % quantile times (t_10_, t_50_, and t_90_) were calculated from the normalized counts versus the time plot, as shown in Fig. 3b.

Induction time in crystallization is defined as the interval between antisolvent addition and the first detectable appearance of crystals. However, in practice, such a definition is highly sensitive to experimental noise, detection thresholds, and the presence of spurious nuclei, which can lead to poor reproducibility. To overcome this, threshold-based definitions such as t_10_ (time until crystal counts reach 10 % of the maximum) or t_50_ (time until 50 % of the maximum is reached) are commonly employed literature [50,51]. While t_50_ includes contributions from crystal growth as well as nucleation, this does not detract from its value as a practical and consistent measure of induction. In fact, t_50_ captures the kinetics of the system in a way that is directly relevant to process reproducibility and scale-up, making it an excellent choice for quantifying induction time in crystallization studies. There is also precedent in literature for using 50 % thresholds to define induction time. Mitchell and Frawley [52] employed a 50 % turbidity rise (t_50_) to determine induction time in cooling crystallization of paracetamol to minimise noise sensitivity. More recently, Cashmore et al.[53] defined isothermal induction time as the duration until optical transmissivity dropped below 50 %. Therefore, t_50_ was used as a measure of the induction time in the current work.

For the continuous antisolvent crystallization experiments, the COBC (547 mL working volume) was initially filled with the antisolvent. All the runs were carried out under the isothermal conditions of 25 °C. The COBC was operated at an oscillation amplitude of 20 mm (10 mm from the centre position) and a frequency of 2 Hz. API solution and antisolvent feed streams were injected through a T-junction at the inlet of the COBC for the COBC case and in the loop (API solution feed followed by antisolvent) for the RC + COBC and VD + RC + COBC cases. The feed streams were injected into the crystallizer or loop at a volumetric flow rate of 21.75 mL/min each (API Solution: 20.31 g/min, Antisolvent: 21.75 g/min) while the product was taken out at 43.5 mL/min. Considering the flow rates and the mass composition of the feed streams, the feed supersaturation at which all the continuous experiments were performed was 0.6. The feed supersaturation ($S_{\mathrm{feed}}$) is defined as the supersaturation at the feed composition, assuming that the feed streams have mixed thoroughly without any crystallization.

(2) $$S_{\mathrm{feed}}=\frac{C_{\mathrm{feed}}}{C_{\mathrm{feed}}^{*}}-1$$

(3) $$C_{\mathrm{feed}}=\frac{Q_{S}C_{S}}{Q_{S}-Q_{S}C_{S}+Q_{\mathrm{AS}}}$$

where $C_{\mathrm{feed}}$ [g Solute/g Solvents] and $C_{\mathrm{feed}}^{*}$ [g Solute/g Solvents] are the solute-free concentration and solubility at the feed composition, assuming that the feed streams are completely mixed. The values of $C_{\mathrm{feed}}$ and $C_{\mathrm{feed}}^{*}$ are 0.044 g Solute/g Solvents and 0.028 g Solute/g Solvents, respectively. $C_{S}$ [g Solute/g Solvents] is the solute-free concentration of paracetamol in the API solution feed stream. $Q_{S}$ [g/s] and $Q_{\mathrm{AS}}$ [g/s] are the mass flow rates of the API solution and the antisolvent feed streams, respectively. The residence time ($\tau_{C}$) inside the COBC was 12.57 min across all the configurations. The residence time inside the flow cell ($\tau_{\mathrm{FC}}$) was 1.84 min, while the recirculation loop (operated at 1.33 LPM) had a residence time ($\tau_{\mathrm{RC}}$) of 2.25 min. The operating conditions across the continuous setups are shown in Table 2.

**Table 2:** Operating conditions for the continuous antisolvent crystallization experiments across different configurations.

**Operating conditions**	**COBC**	**RC + COBC**	**VD + RC + COBC**
Reactor volume (mL)	547	547	547
Flow cell volume (mL)	80	80	80
Loop volume (mL)	−	98	98
Total volume (mL)	627	725	725
Total residence time, τ (min)	14.41	16.66	16.66
RC flow rate (LPM)	−	1.33	1.33

The CLD obtained from FBRM was converted to CSD using the model developed by Honavar et. al.[54], and the FTIR spectra were used to obtain the in-line concentration, C (g Solute/g Solvents) from which the supersaturation ratio, SSR (SSR=S+1) profile was calculated. The FTIR calibration to obtain concentration from the IR spectra is described in **Section S2** of the SI. The dried samples were mounted on aluminium stubs with carbon tape and sputter-coated with gold at 20 mA for 30 s. Then the samples were analysed using a Hitachi SU-70 SEM at 10 kV accelerating voltage, 10 mm working distance, and in field-free mode. Continuous crystallization with the VD + RC + COBC configuration was carried out three times to quantify reproducibility and establish error bars. To minimize solvent and paracetamol usage, experiments with the other two configurations – COBC alone and RC + COBC – were not repeated in the current study. The extent of variability for these configurations is expected to be of a similar order to that observed for the VD + RC + COBC configuration.

## 3 Results and discussion

### 3.1 Influence of HC on the induction time

**Fig. 4:**
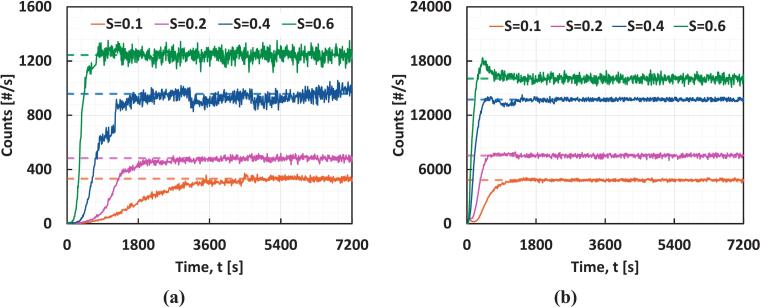
Absolute number counts [#/s] as obtained from FBRM over time **(a)** without VD and **(b)** with VD at different S.

Experiments to measure the induction time were performed at different supersaturations (S=0.1 to 0.6) with and without a VD in the recirculation loop to investigate the effect of HC on the induction time, $t_{i}$. Fig. 4a and Fig. 4b show the FBRM counts during $t_{i}$ measurement experiments with and without VD at different S. At S=0.1, the induction time decreased significantly from 1789 (±39) s to 504 (±16) s.

**Fig. 5:**
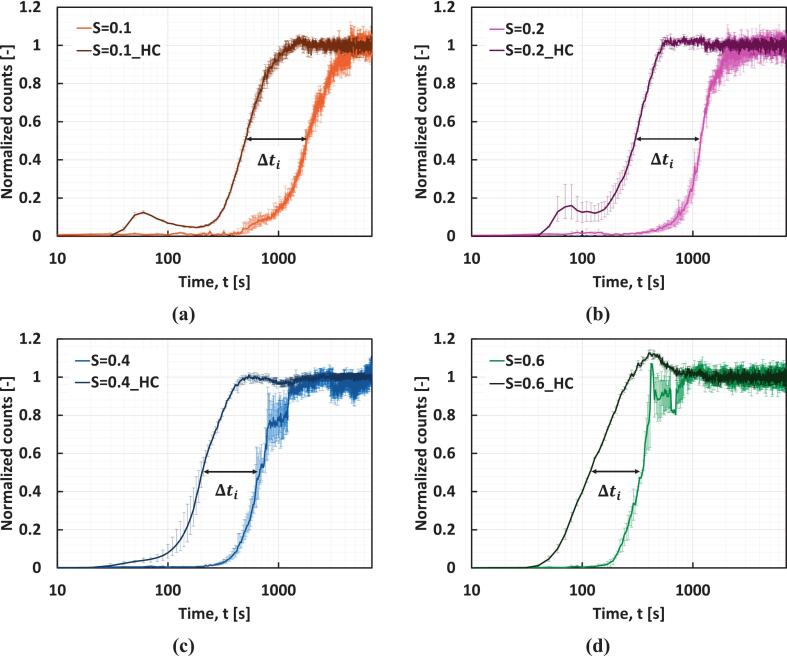
Normalized counts during the induction time measurement experiments with and without HC at **(a)**S=0.1, **(b)**S=0.2, **(c)**S=0.4, **(d)**S=0.6.

Fig. 5 shows normalized counts v/s time at different S for the induction time measurement experiments. In all cases, the induction time decreased significantly under the influence of HC. From the figure, the influence of HC is distinguishable from the change in $t_{i}$ when crystallization takes place under the influence of HC. From Fig. 5a, $\Delta t_{i}$ on using HC at S=0.1 is 1285 s (the average $t_{i}$ reduced from 1789 s to 504 s). The $\Delta t_{i}$ on using HC at S=0.2,0.4, and 0.6 is 874 s, 471 s, and 219 s, respectively (Fig. 5, Fig. 4, Fig. 4). $t_{i}$ from individual runs across different supersaturations is reported in Table 3, with quantifying errors associated with the triplicate run. It can be seen that the effect of supersaturation on $t_{i}$ becomes weaker as supersaturation increases. As discussed earlier in Section 2.3, all these results are based on the experiments conducted at a constant solvent composition (x_w_ = 0.73). On changing the solvent composition from x_w_ = 0.73 to 0.65 at S=0.4, as intuitively expected, $t_{i}$ decreased from 672.6 (±46.6) s to 472.2 s without HC and from 201.8 (±13) s to 176 s with HC. For the sake of brevity, the normalized counts v/s time plot of this is shown in Fig. S4 of the SI.

**Table 3:** Induction time from individual runs across different supersaturations.

**Supersaturation,** S**(−)**	**Induction time,**$t_{i}$**(s)**						${\Delta t}_{i}$**(s)**
	**Run 1**	**Run 2**	**Run 3**	**Average**	**Mean error**	**% error**	
0.1	1743.1	1820.6	1802.5	1788.7	38.75	2.2	1285.1
0.1_HC	488.2	520	502.5	503.6	15.9	3.2	
0.2	1177.6	1175.6	1171.8	1175	2.9	0.2	874.4
0.2_HC	291.7	301.4	308.6	300.6	8.45	2.8	
0.4	692.1	616.3	709.5	672.6	46.6	6.9	470.8
0.4_HC	212.7	206	186.7	201.8	13	6.4	
0.6	313.1	355.1	340.5	336.2	21	6.2	218.8
0.6_HC	115.6	118.2	118.4	117.4	1.4	1.2	

**Fig. 6:**
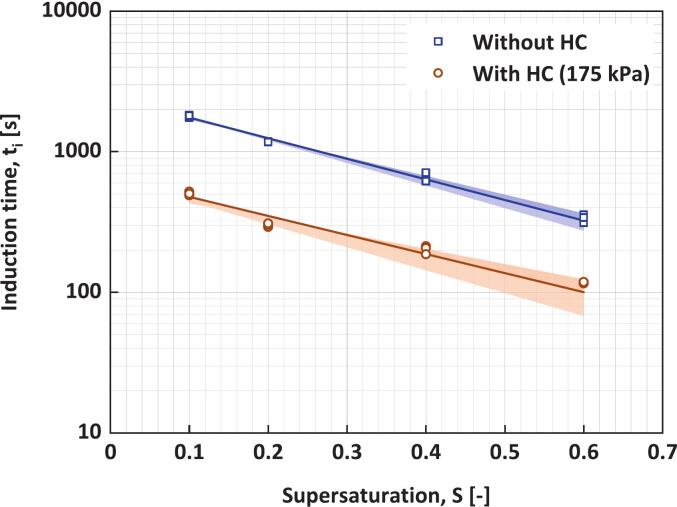
Induction time as a function of supersaturation with and without HC. The symbols are measurements, and the lines correspond to **Equation (4)** with parameters listed in Table 4. The shaded regions denote the 95% confidence intervals of the fitted curves, illustrating the uncertainty associated with parameter estimation.

Effect of supersaturation on $t_{i}$ is more evident from Fig. 6, which shows the induction time as a function of supersaturation with and without HC. The dependence of induction time on supersaturation becomes weaker at higher levels of supersaturation, as observed in previous studies [55,56]. The induction time in the presence of HC is lower than that in the absence of HC at any supersaturation.

Nucleation occurs when molecules collide and aggregate to form molecular clusters and then rearrange themselves within the cluster to form a crystal lattice in a supersaturated solution [57]. Supersaturation in antisolvent crystallization is induced via mixing. HC involves the formation and collapse of cavities in a liquid. The collapsing cavities generate intense shear forces, very high localised energy dissipation rates, and shock waves [33]. These intense shear and dissipation rates improve the mixing of solvent and antisolvent streams. Secondly, HC also provides a heterogeneous surface by creating vapour cavities, which may promote nucleation [30]. The close examination of the experimental data indicates the likelihood of the existence of a finite, minimum induction time ($t_{\mathrm{imin}}$) associated with the nucleation step. The measured induction time ($t_{i}$) data was correlated with supersaturation using the minimum induction time as,

(4) $$t_{i}=\;t_{\mathrm{imin}}e^{\alpha (S_{c}-S)}$$

where $S_{C}$ is the critical supersaturation at which the induction time is minimum ($t_{\mathrm{imin}}$) and α is a parameter describing the sensitivity of induction time with supersaturation. The fitted values of the parameters appearing in **Equation (4)** are listed in Table 4. The method adapted for parameter estimation is detailed in **Section S3** of the SI.

**Table 4:** Parameters of **Equation (4)**.

	$t_{imin}$**[s]**	α**[-]**	$S_{C}$**[-]**
**Without HC**	164 ± 34	3.4 ± 0.3	0.8
**With HC (175 kPa)**	54 ± 22	3.1 ± 0.6	0.8

**Fig. 7:**
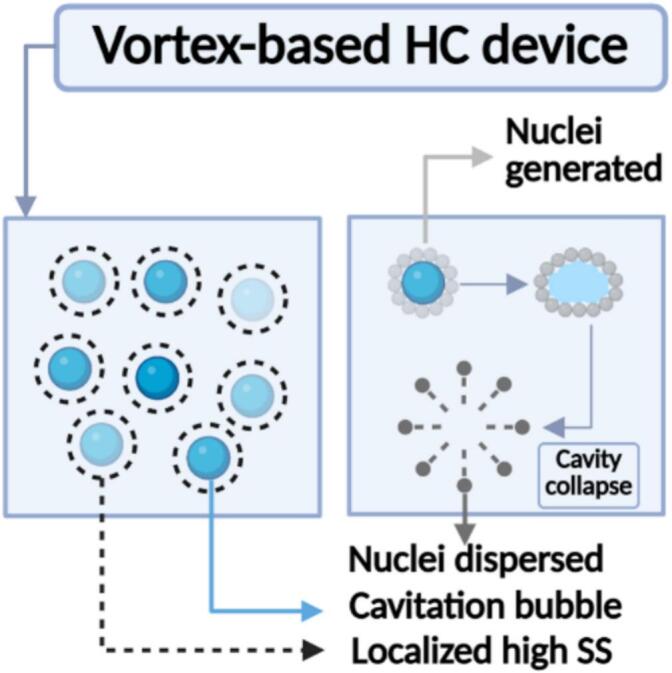
Schematic representation of the cavitation bubbles acting as nucleation sites.

The critical supersaturation used in **Equation (4)** is more or less the same whether HC is applied or not. The intense energy dissipation rates and generation of heterogeneous surfaces via cavities by HC were found to reduce the value of minimum induction time from ∼ 178 s (for without HC) to ∼ 16 s. α was found to decrease when using HC from 3.5 (without HC) to 3. Previous studies have reported that high energy dissipation in supersaturated solutions [58,59,60] and the presence of nano-bubbles [30] improves nucleation. Heterogeneous nucleation occurs in solutions that contain surfaces with a composition different from that of the crystallizing substance − such as crystallizer walls, dust particles, or solid impurities [61]. These foreign surfaces act as active sites for nucleation by lowering the energy barrier required to form nuclei, thereby increasing the local likelihood of nucleation compared to other areas in the system [20]. Hence, it was suggested that the specific foreign surfaces of the cavitation bubbles, as well as the adsorption properties of the forming crystals on the cavitation bubbles, play important roles in the qualitative prediction of the number of induced nuclei. The collapse of cavitation bubbles may disperse the adsorbed nuclei, which can then act as sites for secondary nucleation. Fig. 7 shows a schematic representation of how cavitation bubbles may enhance nucleation by providing additional surface area for heterogeneous nucleation.

**Fig. 8:**
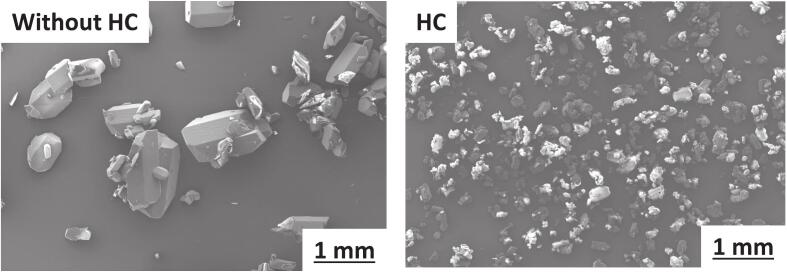
SEM images of the dried crystals obtained with and without HC at S=0.4.

High nucleation rate also affects particle size since more nuclei in the crystallizer grow simultaneously. The SEM images (Fig. 8) show fine particles in the case of HC compared to the case without HC.

**Fig. 9:**
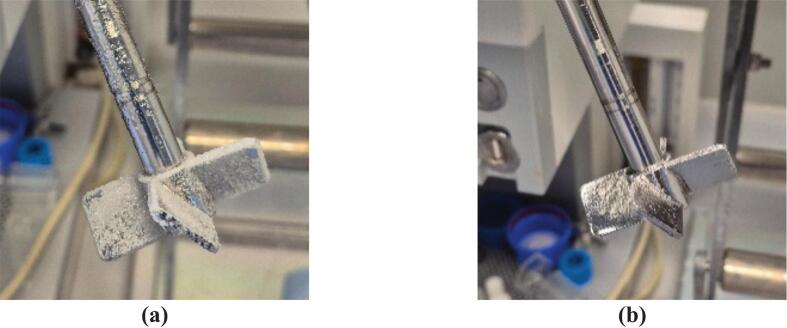
Encrustation on the stirrer during induction time measurement experiments at S=0.6**(a)** without HC, and **(b)** with HC.

Primary nucleation can cause rapid nucleation on the crystallizer walls and result in fouling and encrustation [20]. During the experiments to measure induction time, significant fouling was observed on the stirrer shaft and the impeller in the case without HC. Fig. 9a shows the fouled stirrer shaft and the impeller from the induction time measurement experiment at S=0.6. In the presence of cavitation bubbles, heterogeneous nucleation is enhanced, preventing fouling on the stirrer shaft and the impeller, as shown in Fig. 9b. Without HC, heterogeneous nucleation is predominantly localized on stagnant or low-shear surfaces such as the stirrer shaft and reactor walls, which leads to fouling and encrustation. With HC, the formation and collapse of cavitation bubbles introduce new and significantly larger heterogeneous surfaces (liquid-cavity interface) in the bulk liquid phase, which serve as additional heterogeneous nucleation sites. The availability of additional surface for heterogeneous nucleation reduces surface fouling, as evidenced by the reduced deposition on mechanical components. These observations were consistent across different supersaturations and evidence in support of reduced fouling on the mechanical components by using HC (photographs taken at the end of each experiment) is included in Fig. S5 of the SI.

Significantly reduced encrustation and fouling observed in the case of HC-assisted nucleation will have significant beneficial implications for continuous crystallization. In the case of tubular crystallizers, fouling can lead to clogging, which presents a challenge when using tubular crystallizers for continuous crystallization processes. However, cavitation bubbles can enhance nucleation while preventing encrustation by providing additional surfaces for heterogeneous nucleation away from the crystallizer's walls. The effects of HC, such as faster nucleation rate, less encrustation, less fouling, and reduced particle size, may be beneficial for continuous tubular crystallizers in terms of yield, productivity, and longevity of the continuous process. The application of HC-assisted nucleation was therefore investigated for continuous crystallization, as discussed in the following section (Section 3.2).

### 3.2 Continuous antisolvent crystallization using VD as a nucleator

**Fig. 10:**
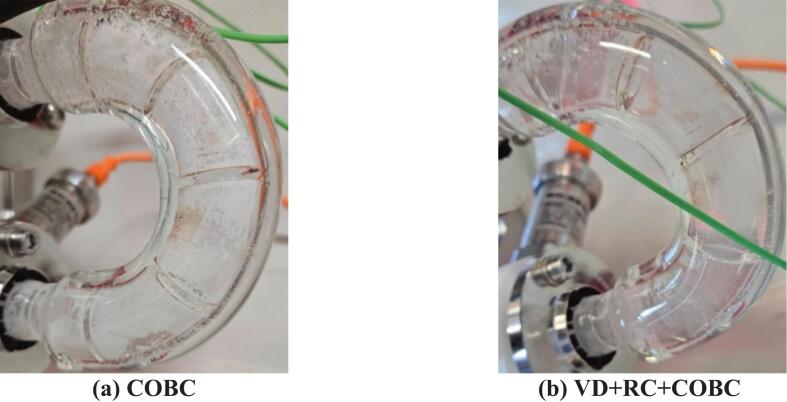
Encrustation on the COBC bends during the continuous antisolvent crystallization **(a)** without HC and **(b)** with HC.

Continuous antisolvent crystallization of paracetamol in a methanol–water solvent system was performed in a tubular crystallizer in three configurations: COBC, RC + COBC, and VD + RC + COBC at S=0.6 to study the effect of HC on nucleation in a continuous crystallization process. While a high primary nucleation rate can enhance yield by generating more nuclei for crystal growth, it can also increase the risk of encrustation on crystallizer walls, potentially leading to clogging and process interruption. The continuous antisolvent crystallization in COBC suffered from encrustation and clogged at t/τ=3.5 (Here τ refers to the total residence time for the configuration – refer to Table 2). Fig. 10a shows the first bend of the COBC during the COBC case experiment, illustrating the solute encrusted on the crystallizer walls. Similarly, the RC + COBC case suffered clogging at *t*/τ = 4.1 at a similar position (at the injection of the API solution stream). The photographs of the clogged location are shown in Fig. S6 of the SI. Using VD in the loop preceding the COBC – VD + RC + COBC resulted in encrustation-free crystallizer walls (Fig. 10b). The VD + RC + COBC case did not suffer from any clogging issues throughout the duration of the experiment (t/τ=11.5). The presence of cavitation bubbles provides heterogeneous nucleation sites, which reduces heterogeneous nucleation on the crystallizer walls, thereby reducing encrustation and improving the longevity of the continuous crystallization process.

**Fig. 11:**
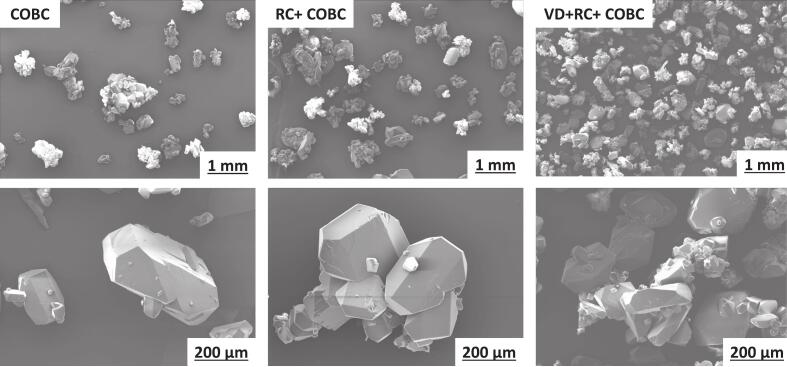
SEM images of the samples taken after steady-state was achieved in the COBC, RC + COBC, and VD + RC + COBC cases.

The SEM images of the crystals produced with VD (shown in Fig. 11) show the presence of smaller crystals attached to or emerging from the surfaces of larger crystals. For the case without VD (the COBC and RC + COBC cases), the crystals were found to have more or less smooth surfaces. The presence of small crystals attached to large crystals seen in the case of VD is unlikely to be because of fragmentation, since in this work, VD was used as a pre-nucleator, unlike the in-line mode used by Tiwari et al.[34] Number distributions of crystals obtained from the presented experiments (Fig. S7) also do not show the presence of any small fragments, since these small fragments are connected to larger crystals and are not counted separately. The different morphology of crystals obtained in the presence of cavitation is likely to be because of the small-scale defects on the generated nuclei due to the collapsing cavities. Detailed investigation of the underlying mechanisms of the different morphologies of the produced crystals in the presence of VD is outside the scope of this work and will be pursued separately.

**Fig. 12:**
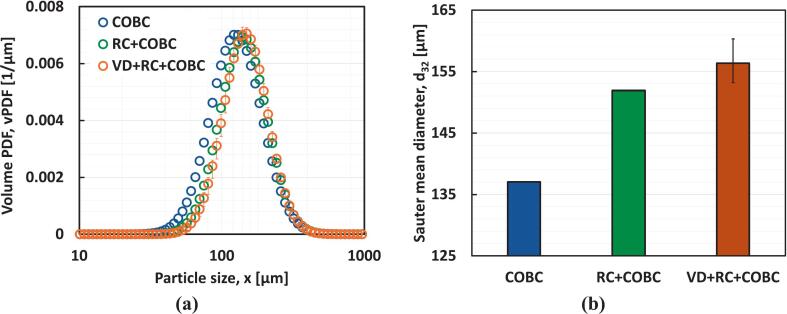
**(a)** Probability density function of volume distribution, and **(b)** Sauter mean diameter, $d_{32}$ at the steady state for COBC, RC + COBC, and VD + RC + COBC.

The effect of HC-assisted nucleation on the overall performance of the continuous crystallizer was then examined. The influence on size (Sauter mean diameter) and distribution (CSD) of paracetamol produced (after achieving the steady state) using the three configurations of continuous crystallizers is shown in Fig. 12a. It can be seen that the use of the nucleator loop preceding the COBC was found to shift the CSD towards the right. The Sauter mean diameter ($d_{32}$) for the RC + COBC case was found to increase from 137 µm observed for the case of COBC to 152 µm, as shown in Fig. 12b. For the case of RC + COBC, $d_{32}$ increases to 152 µm due to the additional residence time provided by the recirculation loop, which provides more time for each particle to grow within the crystallizer. In the case of VD + RC + COBC, $d_{32}$ (156 ± 4 μm) is more or less similar to that for RC + COBC despite the enhanced nucleation rates observed in the presence of VD. The enhanced nucleation in the VD + RC + COBC configuration generates greater number of particles compared to RC + COBC, as confirmed by the higher number of counts detected by FBRM (data not shown here). However, the higher surface area associated with enhanced nucleation (i.e., a larger second moment) also leads to a higher effective growth rate, which depends on the second moment [34]. Consequently, the VD + RC + COBC configuration exhibits higher solute recovery (only 10 % of solute remaining in the mother liquor compared to 26 % for RC + COBC – discussed later in this section). As a result, the Sauter mean diameters for both cases are similar, despite the higher nucleation rate in the presence of VD.

**Fig. 13:**
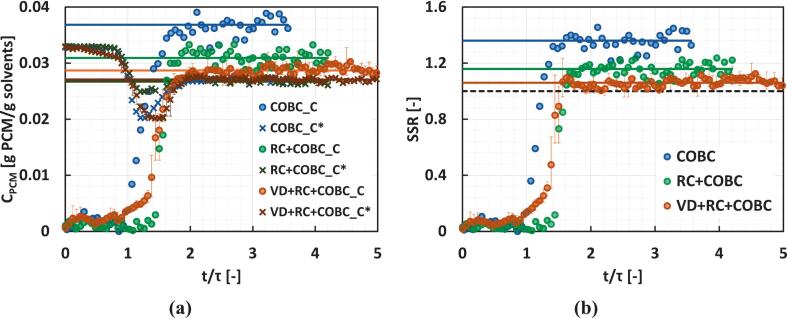
**(a)** Concentration and **(b)** SSR profiles for the continuous antisolvent crystallization of paracetamol.

The transient concentration and solubility profiles for the three continuous crystallizer configurations considered in this work are shown in Fig. 13a and the SSR profiles are shown in Fig. 13b. At the steady state, the COBC case shows the most supersaturation leftover (indicating the lowest yield among the three crystallizer configurations). The RC + COBC case shows a lower steady-state SSR compared to the COBC case. This could be due to the additional residence time provided by the recirculation loop ($\tau_{\mathrm{RC}}=2.25$ min). The residence time of the VD + RC + COBC case is the same as that of the RC + COBC case. However, the steady-state SSR in the case of VD + RC + COBC is even lower compared to that of RC + COBC. Therefore, the further decrease in the steady state SSR must be due to enhanced nucleation rate due to HC, as observed in the induction time measurement experiments.

Based on these results, the steady state uncrystallized solute % and productivity are calculated using the following equations:

(5) $$\mathrm{Uncrystallized}\;solute\;\%=\frac{\mathrm{Theoretical}\;Yield-Actual\;Yield}{\mathrm{Theoretical}\;Yield}=\frac{S}{S_{\mathrm{feed}}}\times 100$$

(6) $$\mathrm{Productivity}=\frac{\mathrm{mass}\;of\;paracetamol\;collected\;(g)}{\tau \;\left(hr\right)\times V_{C}\;\left(L\right)}$$

where $V_{C}$ is the volume of the crystallizer, τ is the total residence time of the crystallizer, and $S_{\mathrm{feed}}$ is the feed supersaturation, which is 0.6.

**Fig. 14:**
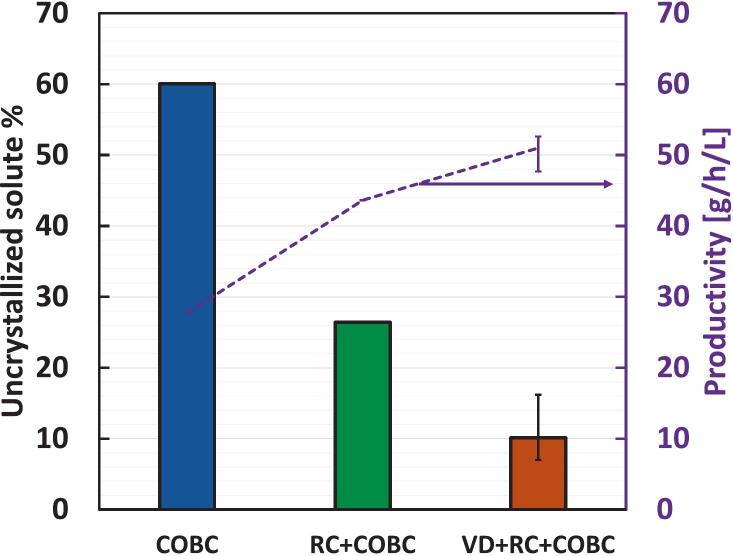
Uncrystallized solute % (bars, left axis) and productivity (dotted line, right axis) for the continuous crystallizer configurations (COBC, RC + COBC, and VD + RC + COBC). The dotted line indicates the trend of productivity.

Fig. 14 shows the uncrystallized solute % and the productivity at the steady state for the three configurations. The COBC configuration resulted in 60 % of the solute leaving the crystallizer in the liquid phase with the mother liquor. Using the recirculation loop (RC + COBC) resulted in less uncrystallized solute (26 %) in the mother liquor due to the additional residence time of the loop. On integrating VD in the loop (VD + RC + COBC), the uncrystallized solute % dropped to 10.1(±4.6) %, indicating almost complete recovery of the solute from the mother liquor and the highest productivity among the three configurations. The VD + RC + COBC case results in an increase in the productivity from 28 (g/h)/L in the case of COBC to 51 (g/h)/L. A previous study on enhancing nucleation to improve the productivity of continuous crystallization of paracetamol by injecting gas bubbles shows a maximum productivity of 16.3 (g/h)/L [62]. Another previous study on continuous crystallization of paracetamol in a tubular slug flow crystallizer presented more than 50 % uncrystallized solute passing through the crystallizer at a similar feed supersaturation in the present work, but used a residence time of ∼ 80 min (∼5 times the τ used in the present work)[63]. Raval et al. (2020)[64] developed a cascaded moving baffle oscillatory crystallizer (CMBOC) for the continuous crystallization of paracetamol and managed to obtain 15 % uncrystallized solute passing through the reactor at τ=20 minutes. Compared to previous studies, the VD + RC + COBC case shows excellent performance with respect to remaining uncrystallized solute (10 %) and productivity [51 (g/h)/L].

The use of a VD-based nucleator preceding the COBC improved the productivity compared to the conventional COBC by ∼ 84 % while reducing encrustation, which increased the longevity of the continuous process by at least 100 % (no clogging till the entire duration of the experiment). The results presented in the current work show the potential of HC as a nucleator and will be useful for designing continuous nucleators and intensifying the continuous crystallization process.

## 4 Conclusions

The current study demonstrates that HC enhances the nucleation rate in the antisolvent crystallization of paracetamol. The effect of HC on the nucleation rate was quantified using induction time measurements, and a correlation was developed to estimate induction time as a function of supersaturation. The effect of HC on the nucleation step was identified, and then the HC device was used as a pre-nucleator using a loop configuration with a continuous tubular crystallizer, COBC. The key conclusions from the present work are:

• HC was found to enhance nucleation and thereby reduce the induction time significantly. At low supersaturation (S=0.1), HC was found to reduce induction time from ∼ 1789 s to ∼ 504 s. At higher supersaturation (S=0.6), the induction time was reduced from ∼ 336 s to ∼ 117 s.
• The influence of supersaturation and HC on induction time may be estimated using **Equation (4)**, comprising a critical supersaturation, minimum induction time for supersaturation beyond critical supersaturation, and a parameter representing sensitivity with respect to supersaturation. The value of critical supersaturation was found to be 0.8, with or without HC. HC was found to reduce the minimum induction time from ∼ 164 s to ∼ 54 s. The parameter representing the sensitivity of induction time with respect to supersaturation was found to decrease slightly from without HC case (3.4 ± 0.3) to with HC case (3.1 ± 0.6).
• The presence of HC was found to significantly reduce encrustation and fouling on the stirrer shaft during batch experiments and on the crystallizer walls in continuous crystallization. The use of a VD-assisted pre-nucleator with the COBC improved the longevity of the continuous crystallization process by at least 100 % by reducing encrustation on the crystallizer walls.
• The VD-assisted pre-nucleator substantially reduced the unrecovered paracetamol in the mother liquor (10 % compared to 60 % for COBC without pre-nucleator). The overall productivity of the continuous crystallizer was enhanced by ∼ 84 % compared to a conventional COBC [from 28 (g/h)/L to 55 (g/h)/L]. The use of a VD-assisted pre-nucleator also increases the Sauter mean diameter from 137 µm to 156 µm.

The results of VD-based HC were found to be very promising in terms of enhanced nucleation, reduced fouling, and enhanced productivity of crystallization. The presented results will be useful in developing and designing process intensification strategies for continuous crystallization using HC.

## CRediT authorship contribution statement

**Vidit Tiwari:** Writing – original draft, Visualization, Validation, Methodology, Investigation, Formal analysis, Data curation. **Subhrajit Swain:** Writing – original draft, Visualization, Validation, Methodology, Investigation, Formal analysis, Data curation. **Vivek V. Ranade:** Writing – review & editing, Supervision, Project administration, Methodology, Funding acquisition, Conceptualization.

## Declaration of competing interest

The authors declare no known competing financial interests or personal relationships that could have appeared to influence the work reported in this paper.
