# Supplementary material for: Enhancing Nucleation using a Vortex-based Hydrodynamic Cavitation Device: Application to Antisolvent Crystallization of Paracetamol – Methanol – Water System
Source: Ultrason Sonochem. 2025 Nov 11;123:107668. doi: 10.1016/j.ultsonch.2025.107668 (PMC12670941; doi:10.1016/j.ultsonch.2025.107668)
Supplement: Supplementary Data 1 [file mmc1.pdf]

SUPPORTING INFORMATION

Enhancing Nucleation Using a Vortex-based Hydrodynamic Cavitation Device: Application to Antisolvent Crystallization of Paracetamol – Methanol – Water System

Vidit Tiwari[1], Subhrajit Swain[1], Vivek V. Ranade[*]
Multiphase Reactors and Intensification Group
Synthesis and Solid-State Pharmaceutical Research Centre
Bernal Institute, University of Limerick, Ireland

[*]Email: Vivek.Ranade@ul.ie

[1]Equal contribution

S1. Experimental setup 2
S2. Calibration of ATR-FTIR 4
S3. Influence of HC on the induction time 5
S4. Continuous antisolvent crystallization using VD as a pre-nucleator 7

S1. Experimental setup

Stirrer speed

The stirrer speed (350 rpm) was set based on the estimation of the critical suspension speed (~310 rpm) required to suspend particles up to 500 μm at a solid loading of 3.6% w/w [30.5 g in 850 g solvents, corresponding to a maximum operating solid loading in the current study – refer to **Table 1** (COMP)], calculated using the Zwietering correlation for critical suspension speed:

$$N_{js} = S.X^{0.13}.d_p^{0.2}.\left[\frac{g(\rho_S - \rho_L)}{\rho_L}\right]^{0.45}.\mu^{0.1}.D^{-0.85} \tag{S1}$$

where, N_{js} [rotations per second] is the critical suspension speed, S is the Zwietering constant, X [g solids/ g liquid] is the mass fraction of the solids (w/w), d_p [m] is the volume mean diameter of the solids, g [m/s^2] is the acceleration due to gravity, ρ_S [1280 kg/m^3] and ρ_L [943 kg/m^3] are the densities of the solid and the liquid phase, respectively. The liquid phase density is calculated by taking a mass-weighted average of the densities of the individual solvents (300g methanol and 550g water). μ is the viscosity of the liquid (9×10^{-4} Pa.s) and D [m] is the impeller diameter (45 mm in the present work). Devarajulu and Loganathan (2016)[1] developed a correlation to calculate the Zwietering constant, S for a 45° pitched blade turbine, which is as follows:

$$S = 10.42\left(\frac{C}{T}\right)^{0.455}\left(\frac{H}{T}\right)^{-0.107} \tag{S2}$$

where C [m] is the clearance of the impeller from the bottom of the vessel (~20 mm), T [m] is the tank diameter (100 mm), and H [m] is the height of the liquid surface from the bottom of the tank (~100 mm). Using Equation S2, S is calculated to be 5.01. Using the Zwietering correlation (Equation S1), N_{js} comes out to be ~270 RPM. The effect of particle size on the critical suspension speed is shown in **Figure S1**. The chosen stirrer speed of 350 RPM is thus enough to create complete suspension of solids but not enough to cause any breakage of crystals.

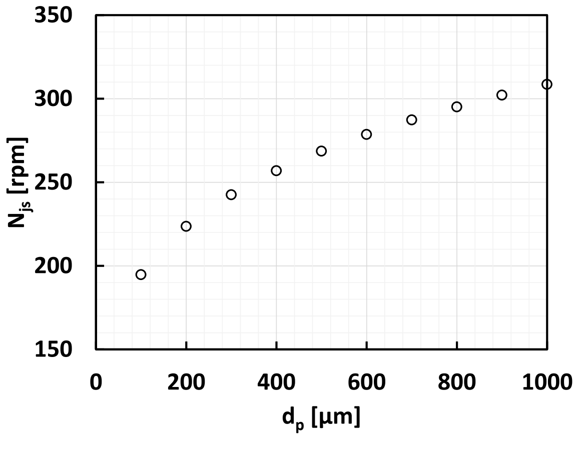

Figure S1. Effect of particle size (mean diameter, d_p) on the critical suspension speed (N_{js}).

Antisolvent addition

The impeller tip speed was calculated using the standard relation $U_{tip} = \pi DN$, where $D = 0.045\,m$ is the impeller diameter and $N = 5.8\,rps$ (350 RPM) is the stirrer speed, yielding a tip speed of 0.8 m/s. Based on the local velocity field (assuming 50% of the tip speed for a 45° downward-pitched blade impeller in a continuous stirred tank crystallizer[2], $U = 0.4\,m/s$) and the cross-sectional area (A) of the dip tube (ID: 9 mm), the volumetric flow rate ($Q = U \times A$) was estimated to be 1.5 L/min. To achieve this, a 400 mL AS dose was added across all the experiments (**Table 1**), using an appropriately programmed Longer L100-1F Intelligent Pump (Precision Fluid Transfer) with YZ1515x head and Longer #18 silicone tubing (ID: 7.9 mm, OD: 11.1 mm), corresponding to a feed time of ~16 seconds. The pump was calibrated before carrying out the actual experiments, and the volume delivered was measured to quantify errors (400±2 mL). Considering the power number $\left(N_Q = \frac{Q}{ND^3}\right)$ for a 45° downward-pitched blade impeller with $D/T = 0.45$ as ~1[2], the circulation time can be estimated as $t_c = \frac{V}{Q} = \frac{\pi}{4NN_Q}$. Assuming mixing time to be ~10 times the circulation time, the mixing time for such a system can be estimated, which is ~2 seconds. Therefore, the higher limit of the supersaturation (S) range was decided where the induction time was at least an order of magnitude greater than the feed and mixing time. The lower range of S was selected such that the corresponding measurements are above the instrumental detection limit.

Labelled photographs of the setup

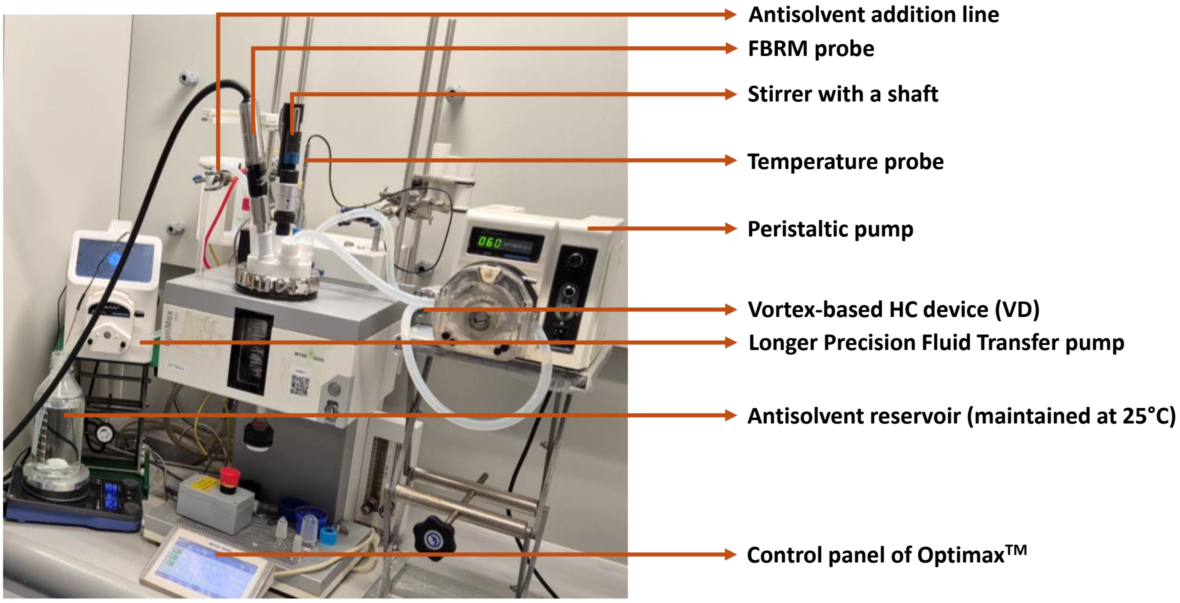

(a)

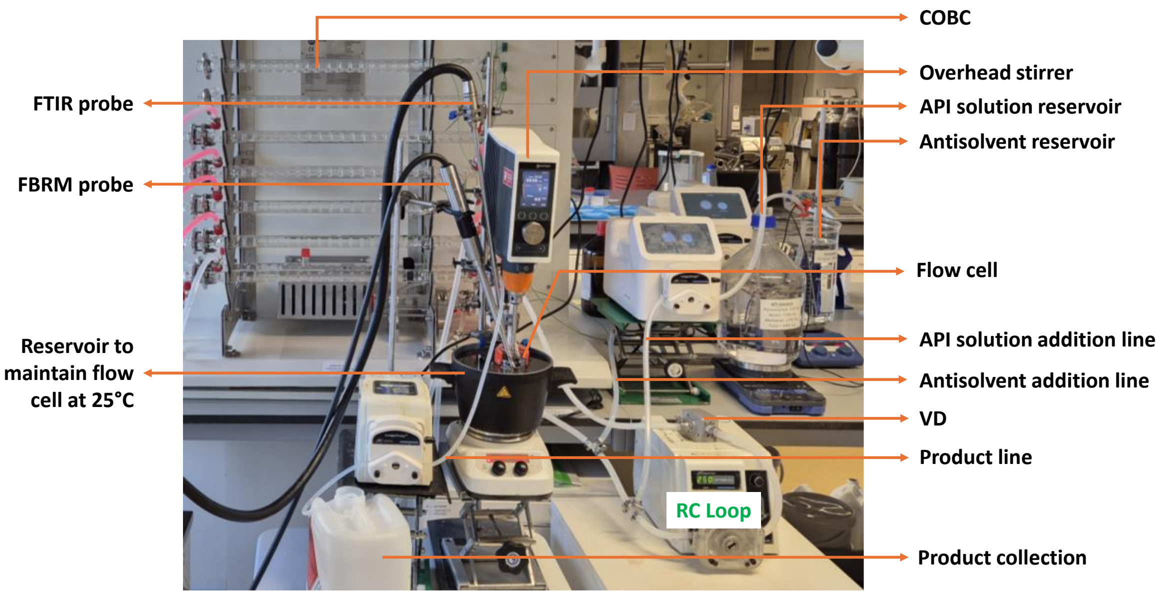

(b)

Figure S2. Labelled photographs of the experimental setup to investigate the effects of HC on **(a)** induction time of crystallization of paracetamol, and **(b)** continuous antisolvent crystallization of paracetamol in a VD+RC+COBC configuration.

S2. Calibration of ATR-FTIR

An ATR-FTIR (ReactIR 15, Mettler Toledo^{TM}) equipped with a 9.5 mm "DiComp" immersion probe with a diamond crystal and silver halide (AgX) optical cable was used to record liquid phase infrared spectra of the crystallizing mixture. The ATR crystal allows the acquisition of the spectra even in the presence of solids. The infrared spectra are known to be affected by the concentration of the solute and the solvent composition. When calibrated with the solvent composition and solute concentration, an ATR-FTIR probe can measure the solute concentration in situ. The amide functional group within the paracetamol molecule emits a bending frequency of 1517 cm^{-1} in Infrared spectroscopy (**Figure S3a**). The calibration procedure used in this work involves tracking the height to a two-point baseline. A two-point baseline was chosen because the absolute peak height may change if the source intensity of the Infrared changes. Nineteen calibration standards were prepared to cover a range of solute-free antisolvent compositions of $x_w = 0.63$ to $x_w = 0.83$, and the solute concentrations from $C = 0$ to 1 *g solute/ g solvents*. Note that the solute concentrations reported here are on a solute-free basis. The solubility data of paracetamol in a methanol-water system, required to prepare the calibration standards, are reported in the literature[3]. The calibration was performed at three different source intensities to confirm that the measurements were independent of disturbances in the source. The values of absorbance (1517 cm^{-1} peak height from two-point baseline) and concentration (g Solute/ g Solvents) were fitted using a second-order polynomial as follows:

$$C = 0.423A + 1.2A^2 + 0.026Ax_w \quad \text{(S3)}$$

Figure S3b shows the calibration curve used in the present work.

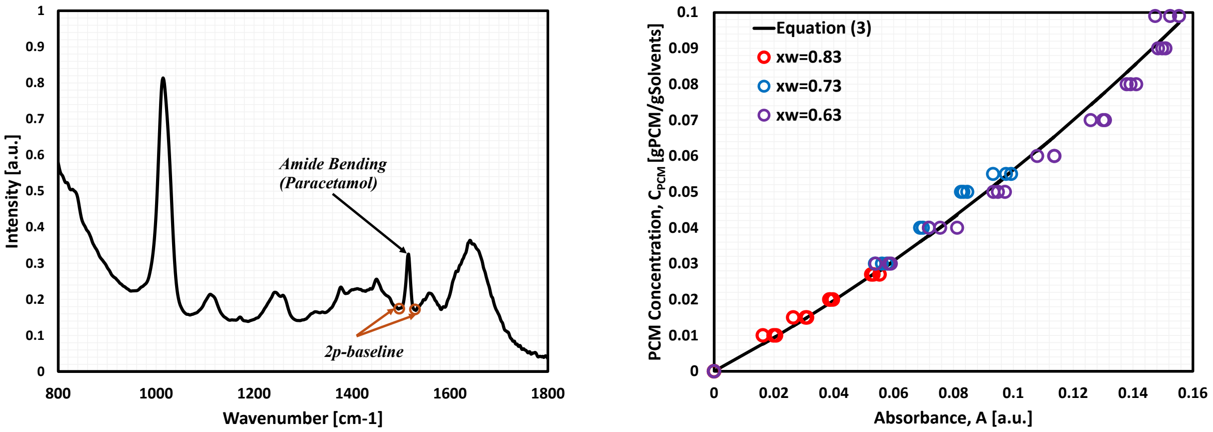

(a) **(b)**

Figure S3. (a) Typical ATR-FTIR Spectra used in calibration, **(b)** Calibration curve, and the calibration standards at solute-free antisolvent mass fractions of 0.63, 0.73, and 0.83. Three measurements were performed at each concentration.

S3. Influence of HC on the induction time

Influence of changing solvent composition on the induction time

On changing the solvent composition from x_w=0.73 to 0.65 at $S = 0.4$, t_i decreased from 672.6 (±46.6) s to 472.2 s without HC and from 201.8 (±13) s to 176 s with HC. The normalized counts v/s time plot of this is shown in **Figure S4**.

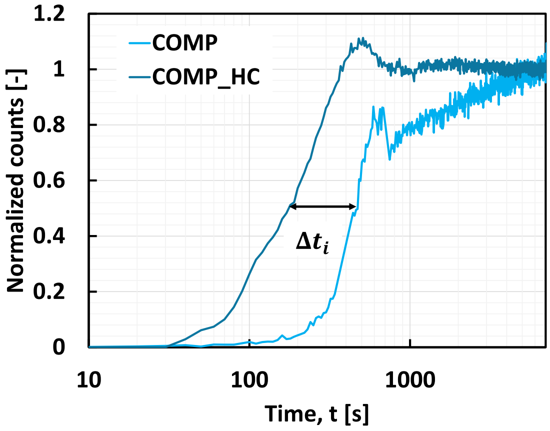

Figure S4. Normalized count v/s time plot on changing the solvent composition from x_w=0.73 to 0.65 at $S = 0.4$ (Experiment name: COMP) – with and without HC.

Parameter estimation for the correlation developed in Equation (4)

Initially, a non-linear optimization using the GRG solver in MS Excel with non-negative unconstrained variables was carried out for both the datasets (HC and without HC). These results indicated $S_c = 0.8$. This value is physically reasonable for such a system and lies above the experimentally studied supersaturation range. With S_c fixed at 0.8, the remaining parameters t_{imin} and α were re-estimated using the nlinfit tool of MATLAB, with initial guesses based on the experimental minimum induction

time and a unit value for α. The regression was performed without applying explicit bounds, and the residuals and Jacobian from nlinfit were used to compute 95% confidence intervals (CI) via nlparci. This strategy was used to obtain CI for the parameters as listed in **Table 4**.

Reduced fouling on the mechanical components

The fouled mechanical components from the induction time measurements without HC were consistent across different supersaturations. The availability of additional surface for heterogeneous nucleation reduced surface fouling while using HC. These observations are shown in **Figure S5**.

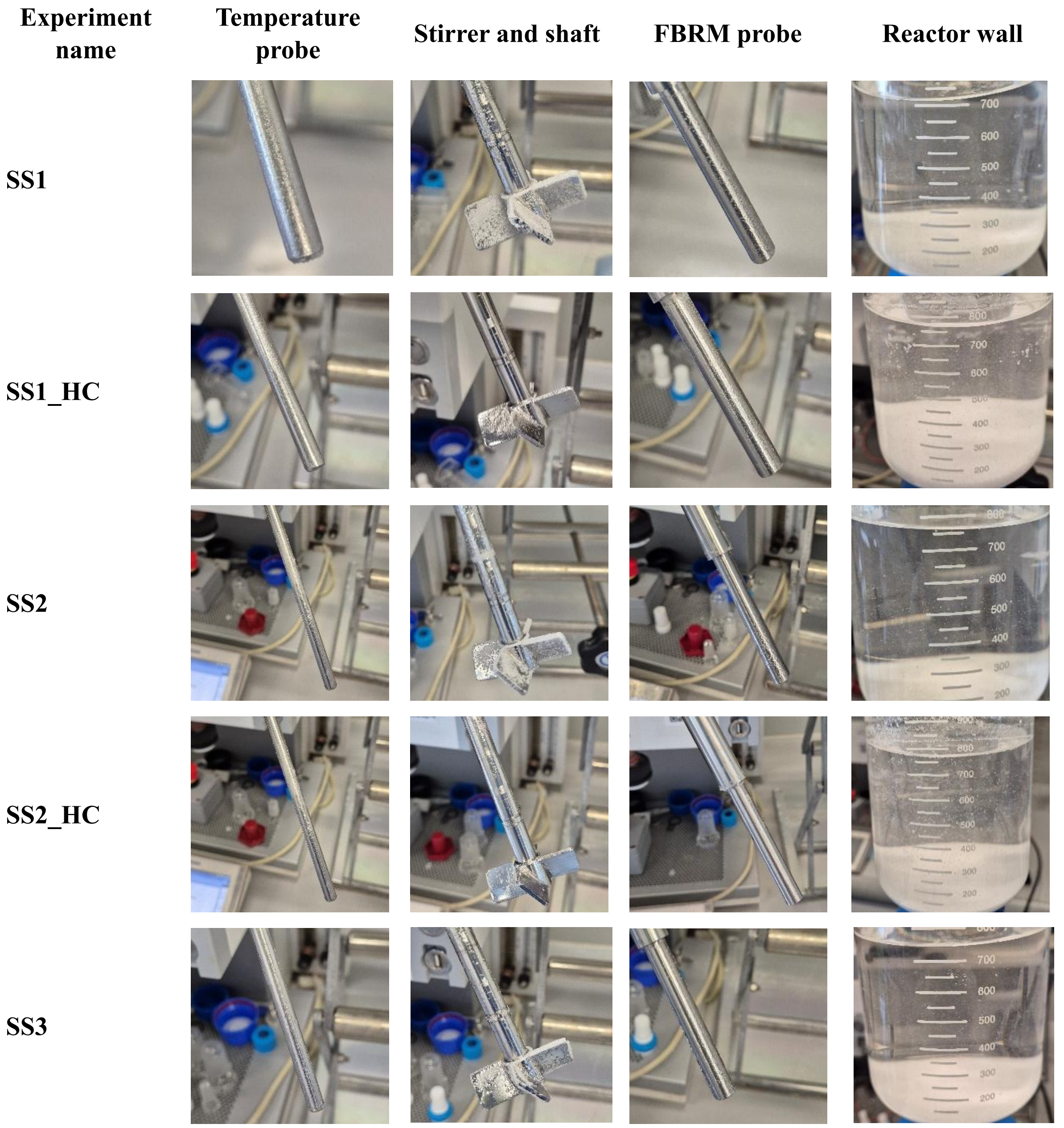

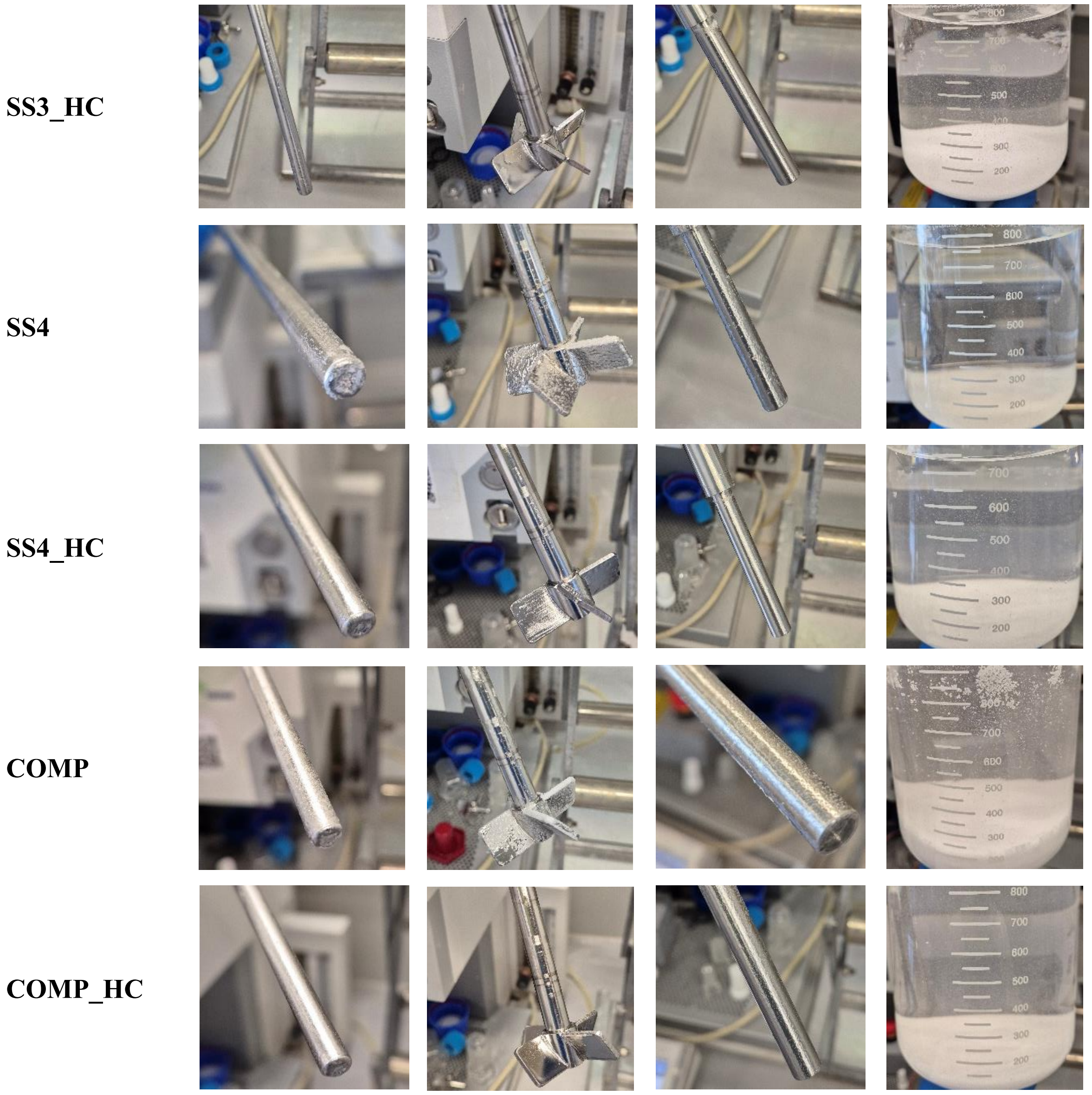

Figure S5. Photographs in support of reduced fouling on the mechanical components by using HC across a range of supersaturations. The photographs were taken at the end of each experiment under similar lighting conditions in the fume hood and used as it is.

S4. Continuous antisolvent crystallization using VD as a pre-nucleator

Clogging in COBC and RC+COBC

COBC suffered clogging at the injection of the API stream at $t/\tau = 3.5$. Similarly, the RC+COBC case suffered clogging at $t/\tau = 4.1$ at a similar position. The photographs of the clogged location are shown in **Figure S6**.

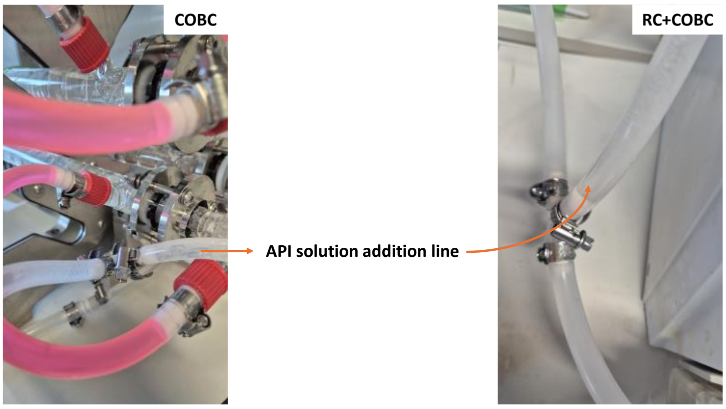

Figure S6. Location of clogging in the COBC and RC+COBC cases.

Number distribution of the crystals

The occurrence of small crystals attached to larger ones in the VD+RC+COBC case (**Figure 11**) is unlikely to result from fragmentation. In this study, the VD functioned as a pre-nucleator rather than being operated in an in-line configuration. Furthermore, the number-based crystal size distributions from different configurations (**Figure S7**) do not indicate the presence of detached small fragments, as these fine crystals were connected to larger ones and therefore were not counted as separate particles.

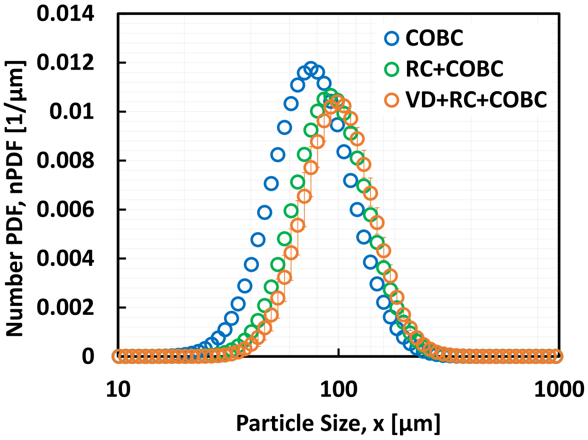

Figure S7. Probability density function of number distribution for COBC, RC+COBC, and VD+RC+COBC cases.

References

1. Devarajulu, C. & Loganathan, M. Effect of Impeller Clearance and Liquid Level on Critical Impeller Speed in an Agitated Vessel using Different Axial and Radial Impellers. *J. Appl. Fluid Mech.* **9**, 2753–2761 (2016).
2. Amanullah, A., Buckland, B. C. & Nienow, A. W. Mixing in the Fermentation and Cell Culture Industries. in *Handbook of Industrial Mixing* 1071–1170 (John Wiley & Sons, Ltd, 2003). doi:10.1002/0471451452.ch18.
3. Ó'Ciardhá, C. T., Frawley, P. J. & Mitchell, N. A. Estimation of the nucleation kinetics for the anti-solvent crystallisation of paracetamol in methanol/water solutions. *J. Cryst. Growth* **328**, 50–57 (2011).
